# Prophylactic treatment of *Glycyrrhiza glabra* mitigates COVID-19 pathology through inhibition of pro-inflammatory cytokines in the hamster model and NETosis

**DOI:** 10.3389/fimmu.2022.945583

**Published:** 2022-09-27

**Authors:** Zaigham Abbas Rizvi, Prabhakar Babele, Srikanth Sadhu, Upasna Madan, Manas Ranjan Tripathy, Sandeep Goswami, Shailendra Mani, Sachin Kumar, Amit Awasthi, Madhu Dikshit

**Affiliations:** ^1^ Immuno-Biology Lab, Infection and Immunology Centre, Translational Health Science and Technology Institute, Faridabad, Haryana, India; ^2^ Immunology-Core Lab, Translational Health Science and Technology Institute, Faridabad, Haryana, India; ^3^ Noncommunicable Disease Centre, Translational Health Science and Technology Institute (THSTI), Faridabad, Haryana, India; ^4^ Pharmacology Division, CSIR-Central Drug Research Institute, Lucknow, Uttar Pradesh, India

**Keywords:** *Glycyrrhiza glabra*, SARS-CoV-2, hamster model, mice and human neutrophil functions, Th2/Th17 differentiation, NETosis

## Abstract

Severe coronavirus disease (COVID-19) is accompanied by acute respiratory distress syndrome and pulmonary pathology, and is presented mostly with an inflammatory cytokine release, a dysregulated immune response, a skewed neutrophil/lymphocyte ratio, and a hypercoagulable state. Though vaccinations have proved effective in reducing the COVID-19-related mortality, the limitation of the use of vaccine against immunocompromised individuals, those with comorbidity, and emerging variants remains a concern. In the current study, we investigate for the first time the efficacy of the *Glycyrrhiza glabra* (GG) extract, a potent immunomodulator, against SARS-CoV-2 infection in hamsters. Prophylactic treatment with GG showed protection against loss in body weight and a 35%–40% decrease in lung viral load along with reduced lung pathology in the hamster model. Remarkably, GG reduced the mRNA expression of pro-inflammatory cytokines and plasminogen activator inhibitor-1 (PAI-1). *In vitro*, GG acted as a potent immunomodulator by reducing Th2 and Th17 differentiation and IL-4 and IL-17A cytokine production. In addition, GG also showed robust potential to suppress ROS, mtROS, and NET generation in a concentration-dependent manner in both human polymorphonuclear neutrophils (PMNs) and murine bone marrow-derived neutrophils (BMDNs). Taken together, we provide evidence for the protective efficacy of GG against COVID-19 and its putative mechanistic insight through its immunomodulatory properties. Our study provides the proof of concept for GG efficacy against SARS-CoV-2 using a hamster model and opens the path for further studies aimed at identifying the active ingredients of GG and its efficacy in COVID-19 clinical cases.

## Introduction

Severe acute respiratory coronavirus-2 (SARS-CoV-2), the causative agent of the ongoing COVID-19 pandemic, has caused more than 6,228,742 COVID-19-related deaths as of 15 May 2022 (https://covid19.who.int/). COVID-19 cases manifest severe pulmonary pathology that is characterized by cytokine release syndrome (CRS), pneumonia, respiratory distress, and prothrombotic state (1–3). In addition, SARS-CoV-2 infection also leads to immune dysregulation characterized by lymphopenia, altered neutrophil response, strong pro-inflammatory response, and elevated levels of reactive oxygen species (ROS) generation (4). These factors together have a role in pathological symptoms characteristic of COVID-19 not only in the lungs but also in other major organs such as the heart, intestine, and brain (5–8). The basis of disease severity is not completely understood; however, factors such as age, dysregulated inflammatory and oxidative stress pathways, and aberrant activation of neutrophils have been implicated. Moreover, a high neutrophil-to-lymphocyte ratio (NLR) and elevated serum levels of IL-8, the neutrophil chemoattractant cytokine, have been shown in severe COVID-19 patients’ deaths (9). Sera and postmortem lung biopsies from COVID-19 patients have a high concentration of neutrophil extracellular trap (NET) components especially in the inflammatory interstitial lesions and airways (10–12). Moreover, activated neutrophils and NETs are known to promote clotting through direct activation of proteolytic coagulation (11, 13). NETosis is a redox-sensitive phenomenon involving both cytosolic and mitochondrial free radicals. NET formation is a defensive microbicidal phenomenon to defeat invading foreign pathogens, but a loss of its control and persistence during inflammation result in the host tissue damage as seen in rheumatic arthritis, diabetes, sepsis, and COVID-19 (10–12).

Though development and active vaccination strategy against COVID-19 have greatly reduced the global morbidity and mortality related to COVID-19, it has been shown that emerging variants of SARS-CoV-2 could not only escape immunity achieved through immunization but also cause morbidity and mortality (14). Furthermore, limited efficacy of vaccine as seen in the case of immunocompromised individuals such as that in HIV patients remains a future challenge. Strategies to develop antiviral or immunomodulatory drugs that can inhibit virus growth or mitigate COVID-19 pathologies offer an exciting alternative strategy to counteract COVID-19-related morbidity and mortality (15–17). Pools of evidence now suggest that dietary food and herbal extracts used as prophylactic treatment could mitigate pathogenic infection-induced pathologies through antiviral activities or immunomodulatory effects (18–21). Previously published reports based on computational aided docking or *in vitro* assays suggest that herbal medicines may be used as an alternative remedy either alone or in combination with COVID-19 modern medicines (22). More recently, Jan et al. (2021) screened 3,000 agents from traditional Chinese medicines against COVID-19 by using an *in vitro* assay system and suggested extracts of ***Ganoderma lucidum***, ***Perilla frutescens***, etc. to be effective against SARS-CoV-2 (18). However, there are very limited studies that have assessed the efficacy of herbal extracts in animal models of SARS-CoV-2. Golden Syrian hamsters have been routinely used as a model for COVID-19 as the ACE2 receptor of hamsters bear high homology with the human ACE2 receptor, which results in efficient virus infection and pulmonary pathology upon SARS-CoV-2 infection (23–25).

Here, we evaluated the prophylactic efficacy of GG, one of the oldest herbal drugs of Ayurveda (the ancient Indian system of medicine), which is commonly used in Asian and European countries. Different parts of GG, especially its roots, have been used extensively as an alternative or complementary remedy for oxidative and inflammatory diseases because of its several pharmacological effects including antiviral, anti-microbial, anti-allergic, anti-asthmatic, and immunoregulatory owing to the presence of a myriad of alkaloids, polyphenols, terpenes, flavonoids, coumarins, and other phytochemicals (26, 27). Given the prophylactic dosage of GG administered to SARS-CoV-2-infected hamsters, we observed prevention of body weight loss, with a decrease in lung viral load. Further investigation revealed that GG treatment acted as an anti-inflammatory factor and profoundly reduced the expression of pathogenic inflammatory cytokines. In addition, GG treatment also suppressed lung injury and related lung injury markers and most remarkably led to the inhibition of plasminogen activator inhibitor (PAI-1), which is implicated in COVID-19-induced thrombosis. GG anti-inflammatory activity was found to be through direct inhibition of Th1, Th2, and Th17 cell differentiation *in vitro*. Our data on human PMNs and mice BMDNs suggest that GG was able to inhibit NET formation in Telratolimod (TRLM)-primed neutrophils after stimulation with PMA or calcium ionophores (A23187 and ionomycin). This effect may be attributed to the ability of GG to limit NET formation by inhibiting ROS generation and cytokine release. Taken together, we provide the first *in vivo* report to show that prophylactic treatment with GG protects SARS-CoV-2-infected hamsters and show mechanistically that this protection could be due to direct virus inhibition or through potent immunomodulatory activity.

## Results

### Prophylactic treatment of GG exhibits protective efficacy against SARS-CoV-2-infected hamsters

**Figure 1:**
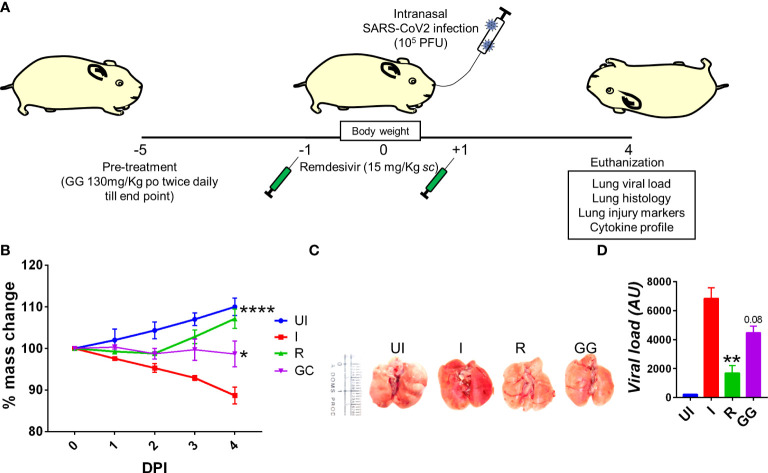
Effect of prophylactic treatment of GG on the SARS-CoV-2-infected hamsters. SARS-CoV-2-infected hamsters were divided into three groups, viz., infection alone (I), infected given remdesivir (R), and infected treated with GG (GG) according to the scheme shown in **(A)**. **(B)** The changes in body mass were recorded each day post infection and plotted as percent change in the body mass; **(C)** representative image of the excised lungs showing regions of inflammation and pneumonitis. **(D)** Relative lung viral load from different groups were estimated by qPCR and shown as bar graph mean ± SEM. *N* = 5 for each experiment. One-way ANOVA using non-parametric Kruskal–Wallis test for multiple comparison. **p < 0.01.

So far, COVID-19 treatment has mostly relied on active vaccination, though a number of potential antiviral drugs and immunomodulatory compounds have shown to mitigate the clinical pathologies of COVID-19 (14). Remdesivir and favipiravir have been shown to manage COVID-19 through direct inhibition of viral replication while drugs such as dexamethasone (DEX) have been shown to act as an immune-suppressant, thereby reducing the strong inflammatory cytokine release and pulmonary damage (16, 17, 28). Some recent studies have pointed out that components of GG such as glycyrrhizin, glyasperin A, and glycyrrhizic acid could be useful against COVID-19 based on computational docking studies (29–32). Moreover, at least two independent groups have shown through *in vitro* studies that glycyrrhizin could suppress SARS-CoV-2 pathology through anti-inflammatory or antiviral properties (29, 30). Based on these reports, we used a hamster model for SARS-CoV-2 infection to evaluate the efficacy of GG in mitigating COVID-19 pathologies. To do so, we followed a 5-day pre-treatment regime prior to challenge, which was continued until end point, i.e., 4 days post infection (dpi) (**Figure 1A**). Golden Syrian hamsters receiving GG showed good recovery in body weight as compared to the remdesivir control (R) hamsters (**Figure 1B**). In line with this, lungs isolated from the euthanized animals on day 4 post challenge showed lower regions of pneumonitis and inflammation as compared to the infected control (**Figure 1C**). Furthermore, we also found a 25%–30% decrease in the relative viral load of the SARS-CoV-2 N2 gene in the GG group as compared to the infected group (**Figure 1D**). Together, our hamster data suggest that prophylactic treatment of GG exhibits protective efficacy against SARS-CoV-2 infection by controlling body weight loss and decreasing lung viral load.

### GG mitigates SARS-CoV-2-induced pulmonary pathology

**Figure 2:**
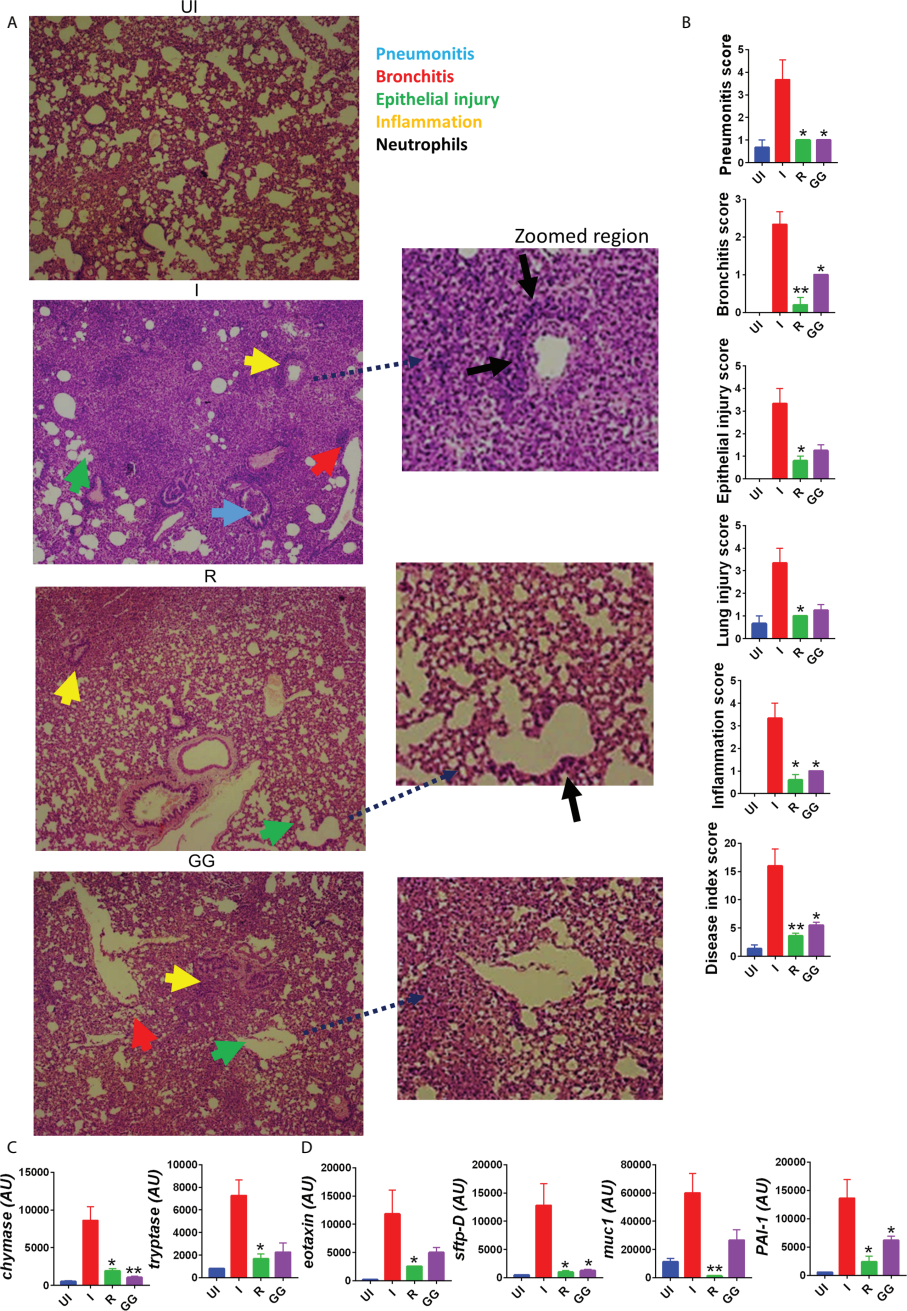
Effect of GG on the lung pathophysiology of SARS-CoV-2-infected hamsters. Left lower lobe of the euthanized hamsters were fixed in formalin, embedded in paraffin, sectioned, and stained with H&E. The H&E-stained slides were then assessed for histological features and scored. **(A)** Representative H&E-stained lung sections of different groups showing pneumonitis (blue), bronchitis (red), epithelial injury (green), and inflammation (yellow) at 10× magnification. **(B)** SARS-CoV-2-infected hamsters were divided into three groups, viz., infection alone (I), infected given remdesivir (R), and infected treated with GG (GG) according to the scheme shown in **(A)**. **(B)** Histological score for pneumonitis, bronchitis, lung injury, epithelial injury, and inflammation as assessed by a trained pathologist. **(C, D)** mRNA expression profile of genes associated with mast cell activation or lung injury, respectively. *N* = 5 for each experiment. One-way ANOVA using non-parametric Kruskal–Wallis test for multiple comparison. *p < 0.05, **p < 0.01.

SARS-CoV-2 infection in clinical cases manifests respiratory distress, which is caused by cytokine release syndromes (3). The aggressive release of cytokine along with cytotoxic immune response in the lungs is associated with pathological lung injury characterized by pneumonitis, alveolar epithelial injury, etc. and elevated levels of injury markers (22, 31). Since prophylactic treatment of GG was able to lower the lung viral load, we tried to understand the efficacy of GG to rescue pulmonary pathologies. We therefore carried out detailed histological analysis at 4 dpi of the isolated lung samples. The HE-stained lung samples of the GG group showed 2/3-fold reduction in the overall disease index score (comparable to remdesivir control group) with profound mitigation in the pneumonitis, bronchitis, alveolar epithelial injury, lung injury, and inflammation histological score as compared to the infection control group (**Figures 2A, B**). One of the important mediators of pathogenic injury of the lung in case of COVID-19 has been identified as mast cells. Our results suggest that GG resulted in the suppression of mast cell functional markers such as chymase and tryptase (**Figure 2C**). Furthermore, we also evaluated the expression of lung injury biomarkers to understand the protection of GG. Prophylactic treatment of GG was found to significantly inhibit the expression of surfactant protein D (sftp-D) and plasminogen activator inhibitor-1 (PAI-1) while reducing the expression of mucin-1 (muc1) and exotoxin to 1.5- to 2-fold as compared to the infection control (**Figure 2D**). Taken together, prophylactic treatment of GG showed recovery in the pathological conditions of the infected lungs with lower expression of lung injury markers.

### GG exhibits potent anti-inflammatory property *in vivo* and limits inflammatory cytokine expression in SARS-CoV-2-infected hamsters

**Figure 3:**
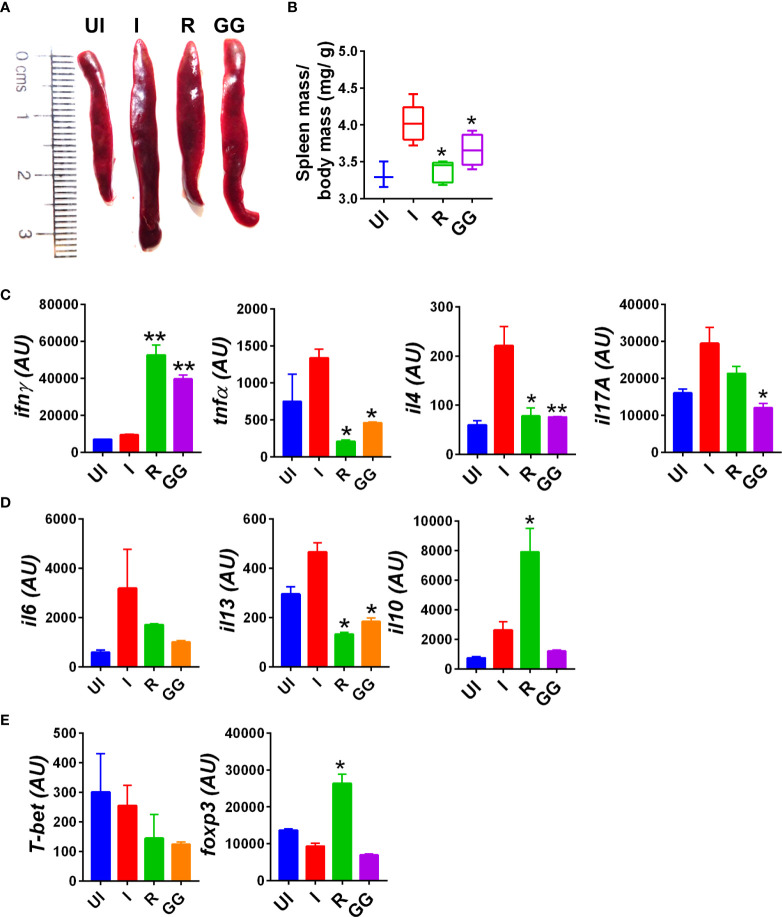
Immunomodulatory effects of GG on SARS-CoV-2-infected hamsters. Immunomodulatory effects of GG were studied in the splenocytes of SARS-CoV-2-infected hamsters and compared with the uninfected control. **(A)** Representative image of excised spleen showing splenomegaly. **(B)** Bar graph showing mean spleen mass/ body mass ratio of each group. mRNA expression of key **(C, D)** inflammatory cytokines and **(E)** transcription factors. *N* = 5 for each experiment. One-way ANOVA using non-parametric Kruskal–Wallis test for multiple comparison. *p < 0.05, **p < 0.01.

Severe clinical cases of COVID-19 have been shown to have elevated cytokine profile, which is known as cytokine respiratory syndrome (3, 33). Heightened levels of pro-inflammatory cytokines along with cytotoxic immune response is a major contributing factor to tissue injury characteristic of severe cases of COVID-19 (34–36). In line with clinical cases, hamsters infected with SARS-CoV-2 show elevated levels of pro-inflammatory cytokine at 4 dpi as previously reported (23, 37). To understand the immunomodulatory effect of GG on SARS-CoV-2-infected hamsters, we investigated splenomegaly condition and evaluated the cytokine and transcription factor expression in the splenocytes. The spleen of the GG group showed alleviation of the hamster’s splenomegaly characteristic of SARS-CoV-2 infection and was comparable to that of the remdesivir group (**Figures 3A, B**). In line with this, the mRNA expression profile of pro-inflammatory cytokines such as IFNγ, TNFα, IL-4, IL-17A, and IL-13 was significantly reduced in the GG group; however, there was no significant changes observed in the mRNA expression of IL-10 and IL-6 cytokines (**Figures 3C, D**). In addition, we also evaluated mRNA expression of Th1 and Treg cells, viz., T-bet and FOXP3, respectively. We did not find any significant changes in the expression of T-bet or FOXP3 in the GG group as compared to the infected control (**Figure 3E**). Taken together, we show that GG, when administered as a prophylactic regime in hamsters, lowers the inflammatory cytokine and thus limits the inflammatory cytokine response, thereby rescuing the hamsters against COVID-19.

### GG suppresses Th1, Th2, and Th17 differentiation

**Figure 4:**
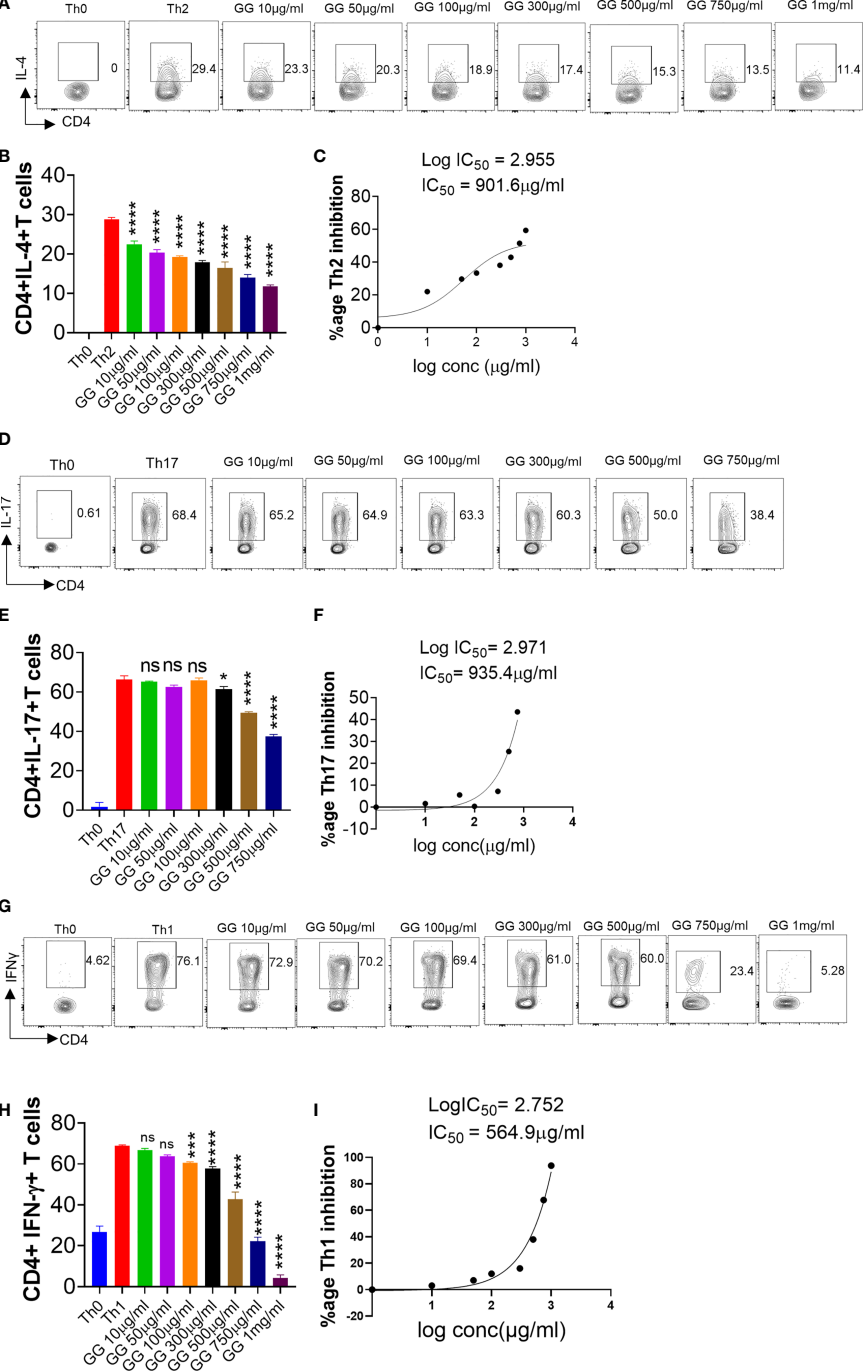
Effect of *Glycyrrhiza gabra* on *in vitro* differentiation of Th1, Th2, and Th17 cells. Spleen and lymph nodes were isolated from 6- to 8-week-old C57BL/6 mice, and their single-cell suspension was prepared. Cells were activated using soluble anti-CD3 antibody and differentiated into helper T (Th)2 **(A, B)**, Th17 cells **(D, E)**, and Th1 conditions **(G, H)** using recombinant mouse IL-4, TGF-β + IL-6, and IL-12 cytokines, respectively. *Glycyrrhiza gabra* was added in concentrations ranging from 10 μg/ml to 1,000 μg/ml at the start of culture. Cells were differentiated for 72 h and IL-4, IL-17, and IFNγ production was measured by intracellular cytokine staining. IC_50_ values were calculated using GraphPad prism software **(C, F, I)**. *p < 0.05, ***p < 0.001, ****p < 0.0001, ns, non significant by one-way ANOVA.

During acute SARS-CoV-2 infection, T cell-mediated adaptive immune response is required for effective viral clearance and generating long-term antiviral immunity (38). However, chronic SARS-CoV-2 infection leads to hyperactivation of T cells and T cell-dependent cytokine release causing immunopathology and poor prognosis (35). Therefore, immune-modulatory drugs are required for the treatment of patients with severe lung inflammation and disease. DEX was used as a positive control since it is a known immunosuppressive drug (**Figure S1**) (28). It was also recommended for COVID-19 patients with severe respiratory symptoms in RECOVERY trial 2020 (28). *In vitro* differentiation of Th1, Th2, and Th17 cells was performed in the presence of graded concentrations of GG and DEX (**Figure 3** and **Figure S1A-I**). DEX inhibited the *in vitro* differentiation of Th1, Th2, and Th17 cells with an increase in the doses (**Figure S1**). The IC_50_ value of Dex was calculated and found to be 1.3 nM, 3.8 nM, and 984 nM for Th2, Th17, and Th1 cells, respectively (**Figures S1C, F, I**). This showed that Dex is a more potent inhibitor of Th2 cell polarization as compared to Th1 and Th17 cells. To study the immunomodulatory role of GG, we studied its effect on *in vitro* differentiation of helper T-cell subsets Th1, Th2, and Th17 cells (**Figure 4**). When incubated with Th2 differentiating cells, GG was able to suppress Th2 cell differentiation profoundly even at a dose of 10 µg/ml (**Figures 4A, B**). Furthermore, the inhibition of Th2 differentiation was found to be concentration dependent, attaining 50% inhibition or IC_50_ at a concentration of 901.6 µg/ml (**Figure 4C**). In contrast, inhibition of Th17 cell differentiation was non-significant at lower concentrations with significant inhibition achieved at 300 µg/ml or above concentration (**Figures 4D, E**). Furthermore, the inhibition of Th17 differentiation was also found to be concentration dependent, reaching 50% inhibition or IC_50_ at a concentration of 935.4 µg/ml (**Figure 4F**). Corroborating the above results, we also found profound inhibition of Th1 differentiation in the presence of GG at 50 µg/ml and above in a concentration-dependent manner (**Figures 4G, H**). The IC_50_ for GG for Th1 differentiation inhibition was found to be 564.9 µg/ml (**Figure 4I**). Together, our *in vitro* data suggest the robust immunomodulatory potential of GG to suppress the differentiation of Th1, Th2, and Th17 cells and thereby might contribute in hamster to suppress the pathogenic inflammatory response post SARS-CoV-2 infection.

### GG exhibited an inhibitory effect on TRLM-PMA/ionophore-stimulated NETosis in human PMNs and murine BMDNs

**Figure 5:**
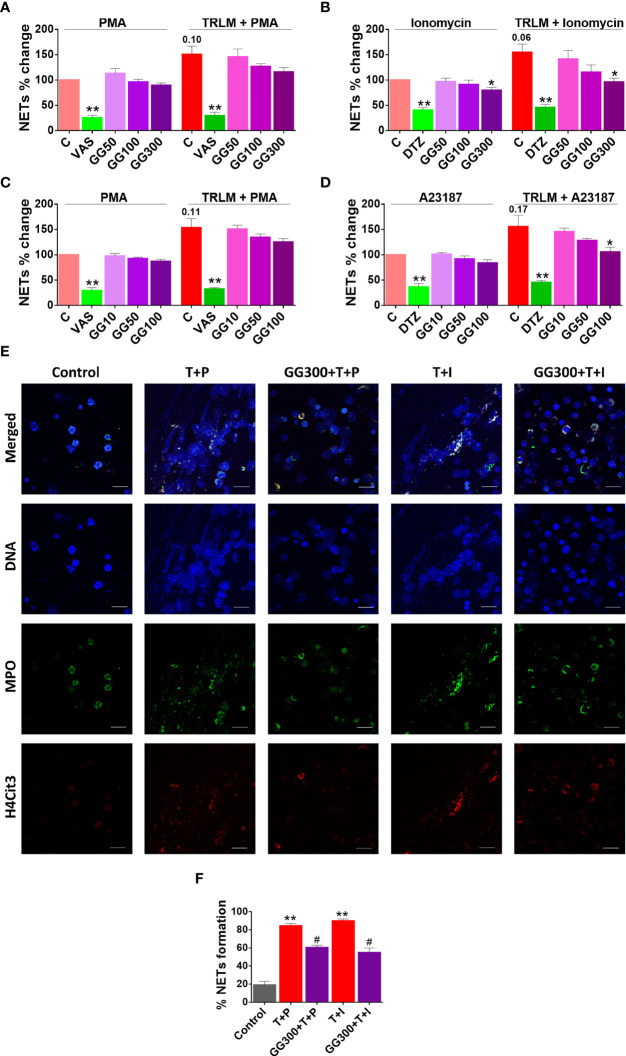
Effect of GG on TRLM-primed PMA/calcium ionophore-induced cytosolic ROS and mtROS production in human PMNs and murine BMDNs. **(A)** Cytotoxic potential of GG extracts on human PMNs and murine BMDNs. Percent cell death was obtained by flow cytometry using PI (10 µg/ml). Doxorubicin (10 µM) was used as a positive control (100%). **(B–I)** After pre-incubation of PMNs and BMDNs with different concentrations of GG, cells were treated with TRLM (10 µg/ml) for 30 min and stimulated with sub-maximal concentration of PMA (12.5 nM) and A23187 (1.25 µM)/ionomycin (2 µM) for 30 min. DCF-DA (10 µM) and MitoSOX (10 µM) were used for cytosolic ROS (**B**, **C**: PMNs; **D**, **E**: BMDNs) and mtROS detection respectively, using flow cytometry. A significant increase in ROS and mtROS were induced by TRLM-PMA/ionophores coupling. GG showed a marked inhibitory effect on PMA and ionophore-induced ROS and mtROS production. NAC and MitoTEMPO were used as the positive control for ROS and mtROS assays, respectively. All the data are represented as mean ± SEM, *n* = min 3 per group, and statistical analysis consisted of one-way ANOVA followed by Bonferroni’s test (*p < 0.05, **p < 0.01 vs. respective control groups; #*p* < 0.05 vs. PMA/ionophore-treated groups). C, control; NAC, N-acetyl cysteine; MT, MitoTEMPO; G10, GG 10 μg/ml; G50, GG 50 μg/ml; G100, GG 100 μg/ml; T, TRLM; P, PMA; I, Ionomycin; A, A23187.

Since GG contains various phytoconstituents with a myriad of activities, we tested the effect of GG for anti-NETotic activity of neutrophils. Much of the understanding of the mechanism that underlines the NOX-dependent and NOX-independent NETosis resulted from the studies using PMA, A23187, and ionomycin (39, 40). We found comparatively more induction of NETs with A23187 in mouse BMDNs and with ionomycin in human PMNs (**Figures S2A–D**), and we therefore used A23187 for mouse BMDNs and ionomycin for human PMNs in the subsequent studies. To assess the effect of TLR7/8 receptor activation, neutrophils were pretreated with TRLM at 10 µM for 30 min, which elicited a significant induction in NETs (51% for PMA and 55% for ionomycin in human, and 52% for PMA and 54% for A23187 in mice) (**Figures 5A–D**). It is important to note that the concentration of PMA and calcium ionophores used here was submaximal (PMA, 12.5 nM; A23187, 1.25 µM; ionomycin, 2 µg/ml). These results show that TRLM or the two types of inducers alone triggered minimal changes, which were increased multi-fold when these were coupled together, indicating the initial priming of TLR7/8 receptors by TRLM and the consequent enhancement of ROS, mtROS, and DNA release by PMA and ionophores.

**Figure 6:**
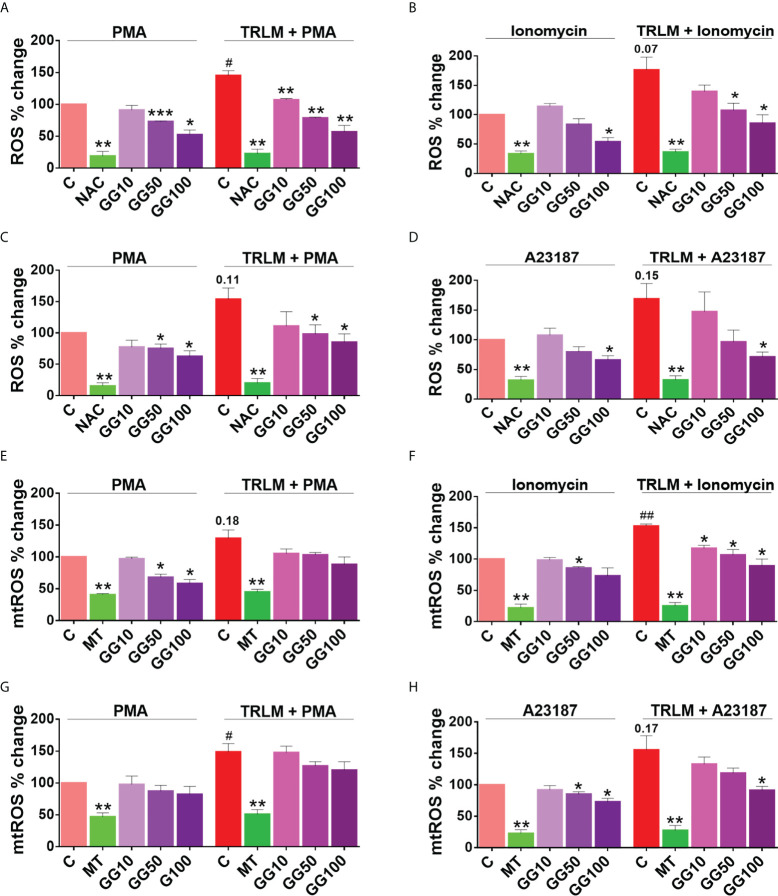
Effect of GG on TRLM-primed PMA/ionophore-induced NET formation in human PMNs and murine BMDNs. After pre-incubation with different concentrations of GG, cells were treated with TRLM (10 µg/ml) for 30 min and stimulated with a sub-maximal concentration of PMA (12.5 nM) and A23187 (1.25 µM)/ionomycin (2 µM) for 30 min. SYTOX Green (100 nM) was used to monitor extracellular DNA release using a plate reader (**A**, **B**, PMNs; **C**, **D**, BMDNs). Total MFI in each experimental condition is expressed as mean ± SEM of four to six experiments. A significant increase in NET formation was induced by TRLM-PMA/ionophores coupling. GG showed a noticeable inhibitory effect on ionophore-induced NETosis. **(E)** NETosis in human PMNs was also monitored using immunofluorescence imaging with DAPI (blue), anti-MPO antibody (green), and anti-H4Cit3 antibody (red). GG showed reduction in nuclear size after induction with ionomycin. Representative fields are shown at 100× with a scale bar of 10 µm. **(E)** Bar diagram representing quantification of percent NETs forming cells as calculated from five transects from three independent experiments. VAS2870 and Diltiazem were used as the positive controls. All the data are represented as mean ± SEM, *n* = min 3 per group, and statistical analysis consisted of one-way ANOVA followed by Bonferroni’s test (*p < 0.05, **p < 0.01, ***p < 0.001 vs. respective control groups; ^#^ *p* < 0.05, ^##^ *p* < 0.01 vs. PMA/ionophore-treated groups). C, control; V, VAS2870; DTZ, Diltiazem; G10, GG 10 μg/ml; G50, GG 50 μg/ml; G100, GG 100 μg/ml; G300, GG 300 μg/ml; T, TRLM; P, PMA; I, Ionomycin; A, A23187.

Currently, approaches to prevent NET generation are considered an attractive strategy to limit uncontrolled inflammation for COVID pathologies in hamster (39). Supplementing the cells with GG could inhibit double-stranded DNA release, a hallmark for NET formation. GG exhibited an inhibitory effect on NETs in a concentration-dependent manner. A low concentration of GG has no significant effect on the inhibition of NETs in neutrophils; however higher concentrations exerted an inhibitory effect on the release of dsDNA (**Figures 5A** and **6B**). Ionomycin-induced NETosis in human PMNs was reduced from 25% to 36% after treatment with 100 and 300 µg/ml, *p* < 0.05 of GG, respectively. In contrast, with TRLM-PMA stimulation, GG did not exert a noticeable reduction in DNA release; a maximum of 20% inhibition was seen at 300 μg/ml. Treatment of TRLM-A23187 stimulated murine BMDNs with 10, 50, and 100 µg/ml GG resulted in downregulation of NETosis to 5%, 18%, and 32%, respectively (*p* < 0.05), while exposure to TRLM-PMA resulted in 13% (50 μg/ml) to 22% (100 μg/ml, *p* < 0.05, **Figures 5C** ,**6D**). **Figure S3A** shows that more than 90% of cells remain viable up to 300 μg/ml in both human and mice. The IC_50_ values of GG on NETs in BMDNs (100.2 and 89.5 µg/ml using PMA and A23187, respectively) were calculated, and all the experiments were conducted using GG concentration based on the calculated IC_50_ values (**Figures S3B, C**).

Moreover, we further confirmed the anti-NETotic effect of GG *in vitro* by immunofluorescence assay. Primed and induced PMNs exhibited a prominent swollen and diffused cell with thread-like structures (**Figure 5E**). In agreement with our fluorimetry data, we found a comparatively greater effect of GG against the calcium ionophore-treated group. **Figures 5E, F** show that the pre-treatment of PMNs with 300 μg/ml GG prevented the diffused and web-like state of TRLM-ionomycin-treated cells as evident by the reduction of the percentage of NET-forming cells, MPO, and H4Cit3 expression. Incubation of GG with the TRLM-PMA group did not result in much elimination of characteristic DNA fiber extrusion, except with shrinkage of nuclear diameter. Results obtained thus indicate that GG might contribute to the regulation of neutrophil NET formation in hamster *via* modulating the ionophore-mediated signaling pathways involved in NETosis.

### GG suppresses ROS and mtROS production

As GG treatment showed a reduction in NET formation in the primed and activated neutrophils from both mice and human, we therefore examined its antioxidant activity in an attempt to decipher its mechanism. When treated with PMA or ionophores before priming with TRLM, a steep rise in superoxide radical production of more than 50% were observed in both the PMNs and BMDNs; ROS (48% for PMA and 75% for ionomycin in human, and 55% for PMA and 70% for A23187 in mice) and mtROS (30% for PMA and 51% for ionomycin in human, and 50% for PMA and 60% for A23187 in mice) (**Figures 6A–H**).

The effect of GG on ROS and mtROS production exposed to TRLM-PMA/ionophores in the presence or absence of GG was measured using flow cytometry. A marked decrease in ROS production in PMNs, from 20% (10 μg/ml), 37% (50 μg/ml), to 50% (100 μg/ml, *p* < 0.05) was observed when stimulated with TRLM-ionomycin, whereas TRLM-PMA led to a decrease of 22% at 10 μg/ml, 45% at 50 μg/ml, and 60% at 100 μg/ml, *p* < 0.05 (**Figures 6A, B**). In BMDNs, GG also had similar effects in limiting the formation of superoxide anions elicited by TRLM-A23187 (13% reduction at 10 μg/ml, 43% at 50 μg/ml, and 58% at 100 μg/ml, *p* < 0.05) or TRLM-PMA (maximum decrease of 45% at 100 μg/ml, *p* < 0.05) (**Figures 6C, D**). The median inhibitory concentration (IC_50_) of GG on ROS in BMDNs was 52.3 µg/ml with PMA and 41.8 µg/ml with A23187 (**Figures S3D, E**).

Similarly, GG concentrations were selected for mtROS assay based on the calculation of its IC_50_ in mice BMDNs; 61.8 μg/ml with PMA and 56.3 μg/ml with A23187 (**Figures S3F, G**). The ability of GG (100 μg/ml) to inhibit mtROS production in PMNs revealed a 40% and 30% reduction with TRLM-ionomycin and TRLM-PMA, respectively (**Figures 6E, F**). A similar percent reduction was also seen in murine cells with TRLM-A23187 (40%) and TRLM-PMA (20%) at 100 μg/ml of GG (**Figures 6G, H**). Notably, GG was comparatively more efficient in reducing calcium ionophore-mediated ROS and mtROS production in neutrophils.

### Effect of GG on pro-inflammatory cytokines production and phagocytosis

One of the hallmark features of COVID-19 is the neutrophilia and cytokine storm that occurs more readily in the lungs as neutrophil numbers are high in pulmonary vasculature than systemic blood vessels (40). We evaluated the degree of inflammation in response to TLR7/8 priming and activation by PMA or A23187 in murine BMDNs, by monitoring the levels of IL-6 and TNFα and their modulation by GG using commercial ELISA kits (**Figures S4A, B**). An increase in the production of IL-6 and TNFα was observed when cells were primed with TRLM before treatment with the inducers: IL-6 (52% in PMA and 78% in A23187) and TNFα (96% in PMA and 45% in A23187). GG (100 μg/ml) exerted a profound inhibitory effect on the release of IL-6 and TNFα from neutrophils when treated with either TRLM-PMA or TRLM-A23187. In particular, IL-6 and TNFα levels were reduced to 33% and 37% (*p* < 0.05), respectively, when exposed to TRLM-A23187, whereas TRLM-PMA treatment resulted in a decrease of 15%–20% in both the cytokines.

Because phagocytosis is one of the important functions of neutrophils to evade any foreign pathogen, we next examined the effect of GG, if any, on this property of the neutrophils. Human PMNs pretreated with GG for 60 min followed by PE-labeled latex beads (1 µm) exhibited a modest reduction [14% (*p* < 0.05)] in the phagocytosis only at the highest concentration (300 µg/ml, **Figure S4C**), while 1–100 µg/ml of GG did not cause any noticeable change. Furthermore, since GG treatment showed a mild inhibitory effect on phagocytosis, we subsequently assessed its effect on the bactericidal activity. Incubation of overnight grown kanamycin-resistant *E. coli* with neutrophils resulted in the reduction of colonies from 7.9 × 10^8^ to 2.4 × 10^8^ CFU/ml (70% reduction, *p* < 0.05, **Figures S4D, E**). Pre-treatment of human peripheral neutrophils with 300 µg/ml GG did not impart any significant effect on the killing activities of phagocytes; approximately 50% reduction in *E. coli* growth was observed when bacteria were incubated with GG-treated PMNs. However, a notable reduction in bacterial growth of 17% (*p* < 0.05) was observed at 300 µg/ml GG, suggesting a direct anti-microbial effect of GG on microbial growth (**Figures S4D, E**).

### Prophylactic use of GG ameliorates COVID-19 pathology in hamster

**Figure 7:**
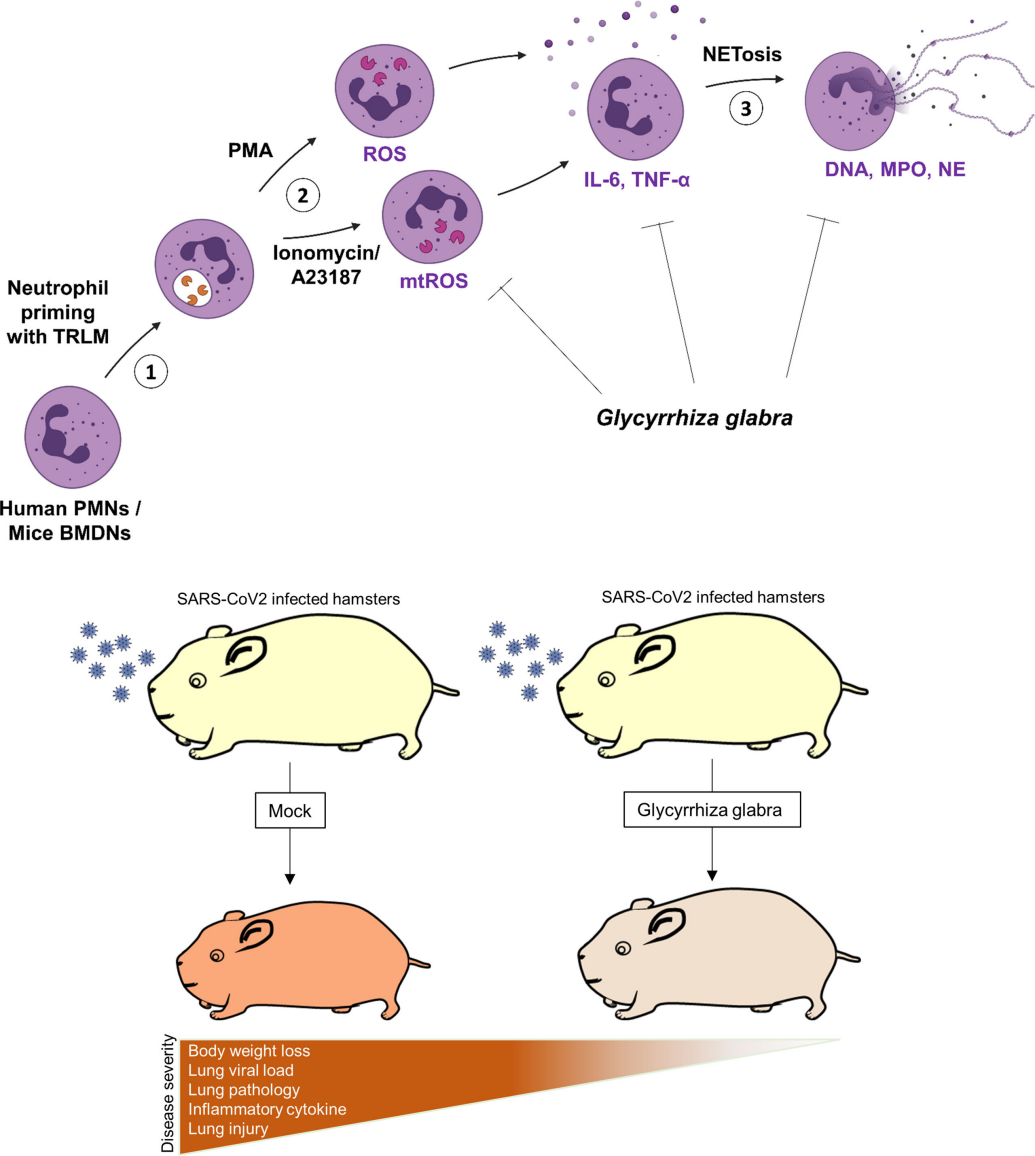
Schematic diagram depicting the protective efficacy of prophylactic treatment of GG on SARS-CoV-2-infected hamsters and a putative mechanism of protection.

In summary, we report that the prophylactic use of GG in SARS-CoV-2-infected hamsters helps in mitigating the COVID-19 pathology. The effect of GG against COVID-19 could be through its potent immunomodulatory potential, inhibitory effects on NETosis, and suppression of ROS generation as illustrated in the summary figure (**Figure 7**).

## Discussion

The recent emergence of SARS-CoV-2 has caused unprecedented mortality, which led to the development and emergency usage of COVID-19 vaccines (https://covid19.who.int/). In addition, therapeutic use of antiviral drugs such as remdesivir (a Rdrp prodrug inhibitor) or favipiravir (an inhibitor of Rdrp) has been shown to decrease viral load and mitigate COVID-19 complications (16, 17). However, novel therapeutics development and clinical trials often end up taking many years, which demands repurposing of the existing compounds. Another exciting alternative therapy is provided by herbal medicines, which are often used as a food supplement in many places in Asia and Europe (18–20). A recent report published in *PNAS* 2021 used 3,000 Chinese herbal extracts from traditional medicine for the high-throughput screening against SARS-CoV-2 *in vitro* (18). The study identified a number of potential herbal extracts that showed remarkable ability to inhibit SARS-CoV-2 entry or replication. *Glycyrrhiza glabra* herbal extract has been traditionally used in Asian countries especially India as herbal tonic. Recent publications on COVID-19 using *in vitro* and *in silico* approaches have shown that components of *glycyrrhiza* such as glycyrrhizin and licorice may exert immunomodulatory activity, which might be beneficial against COVID-19.

In the current study, GG antiviral and immunomodulatory efficacy against SARS-CoV-2 was investigated by using a small animal model of SARS-CoV-2 infection. GG is an Indian traditional herb that is part of ancient Ayurveda medicine (41). GG has been shown to contain various active pharmaceutical ingredients such as glycyrrhizin, liquiritin, isoliguiritin, glyasperin A, and glycyrrhizic acid, and has been shown to have some degree of inhibitory activity against SARS-CoV-2 as shown by computational studies or antiviral screening *in vitro* (29–32, 42). Moreover, a multicomponent study under phase 2 and 3 clinical trials, involving the deglycyrrhizinated form of GG has shown positive results in boosting the host immunity against COVID-19 (43) (ClinicalTrials.gov Identifier: NCT04553705). However, to date, no studies have been conducted to investigate the protective response of GG against SARS-CoV-2 infection by using animal models.

Corroborating the previously published computational results, we found that GG, when given as a prophylactic regime to hamsters, results in substantially reduced gross pathology with a significant prevention of body weight loss. Moreover, the GG group also showed reduction in the lung viral load as compared to the infected group; however, as compared to the remdesivir group, the inhibition by GG was lesser by 10%–15%. Protection in the gross clinical parameters of GG-administered animals prompted us to look at the pathophysiology of lung as COVID-19 clinical cases are characterized by lung injury and pneumonitis. Remarkably, the histological parameters of GG lungs showed alleviation of lung pathology with overall reduced lung injury score and lowered expression of lung injury markers. These results suggested that prophylactic treatment of GG is beneficial against COVID-19 in hamsters, which involves resolving the pulmonary pathology.

Previously published literature on GG or its components have shown robust immunomodulatory effects of GG. For example, licorice, which is a constituent of GG, was able to suppress LPS-induced NLRP3 inflammasomes and NF-kB activation (44). This would mean that GG could lower the inflammatory response induced by LPS. When the mechanism of this inhibition was investigated, it was found that licorice inhibits COX-2 expression and in turn reduces the prostaglandin (PGE2) levels (27, 44). Another study has documented that glycyrrhizin, another component of GG, reduces ROS generation by neutrophils, thereby suppressing tissue inflammation (26, 45). These studies prompted us also to investigate the immunomodulatory potential of GG. When splenocytes’ mRNA expression was studied for cytokines, we found that GG remarkably inhibited pro-inflammatory cytokines in a way that was comparable to remdesivir, a prototype of antiviral drug. Higher levels of cytokines such as TNFα and IL-17 in the early stages of infection have been linked to worsening of the disease and tissue injury. Remarkably, GG was able to suppress the expression of all these three cytokines at the peak of infection in hamster (i.e., 4 dpi). In addition, GG also suppressed the expression of T-bet, which is a key transcription factor for Th1 response. This was an interesting finding, corroborating the immunomodulatory potential of GG previously shown in the context of other pathogenic infection, as it proved that the protective effect of GG against COVID-19 involves, in part, both antiviral activity and immunomodulatory potential resulting in anti-inflammatory response.

CD4+ T helper cells are crucial components of adoptive immunity, which coordinates in providing immunity against pathogenic infection. On the other hand, it has also been shown that dysregulated effector and regulatory T helper cell response leads to tissue injury and worsening of the disease, which is also seen in the case of COVID-19 (36, 46). To further validate the anti-inflammatory potential of GG and to evaluate GG’s effect on suppression of effector T cells, we performed T-cell differentiation assay *in vitro* in the presence or absence of GG. Our results show that GG could inhibit the differentiation of Th1, Th2, and Th17 in a dose-dependent manner much like DEX, a known immunosuppressant (28). Interestingly, the lowest ID_50_ for the *in vitro* assay was found to be for Th1 differentiation, suggesting that GG at lower doses inhibits Th1 differentiation dramatically.

Since GG was also found to significantly inhibit the thrombosis marker PAI-1 and, during COVID-19, induction of thrombosis is strongly correlated with the neutrophil-induced NETosis, we investigated further if GG could modulate the neutrophil response and affect ROS generation. While neutrophils are an important innate immune cells in the context of viral immunity, generation of ROS has been linked to cellular injury leading to pulmonary damage and extra pulmonary organ damage (10). NETs are made up of the dsDNA fibers extruded from neutrophils, containing citrullinated histones and granular enzymes, such as myeloperoxidase (MPO), neutrophil elastase (NE), cathepsin G, α-defensins, bactericidal permeability-increasing factor (BPI), and pentraxin 3. Agonists of endosomal TLR 7/8, which bind to viral single-stranded RNA as in SARS-Co-2 and influenza virus, have been shown to induce neutrophil activation; however, little is known about a putative link between TLR7/8 signaling and the release of NETs in neutrophils. In the present study, TRLM upregulated PMA, and calcium ionophores induced neutrophil functions, indicative of its priming effect. Though the effect of TRLM on oxidative stress in neutrophils has not been elucidated before, similar agonists such as resiquimod (R848) and its water-soluble derivative CL097 have been found to prime the neutrophils and induce cytokines and ROS production (47–49). The molecular basis of such enhancement of neutrophil-derived functions is still lacking; however, priming of TLR7/8 receptors enhances the survival of neutrophils and also augments neutrophil functions (50–52).

Involvement of TLRs for the release of NETs has already been described earlier; Awasthi et al. (2016) had reported that blocking TLR-2 and -6 with specific antibodies significantly reduced oxLDL-induced NET formation (53). Our *in vitro* data highlighted that NETs released in TRLM-primed PMNs following treatment with PMA and calcium ionophore were reduced by GG pretreatment in a concentration-dependent manner. The amount of DNA release in neutrophils stimulated with calcium ionophores showed a more robust reduction as also evident by immunolabeling of MPO and citrullinated histones in the GG-pretreated cells. There are no reports on the effect of GG on NETs; however, other medicinal herbs such as Danshen, the dried root of *Salvia miltiorrhiza*, and the compounds like salvianolic acid B and 15,16-dihydrotanshinone have been found to be effective in reducing NETs by inhibiting MPO and NOX (54). Extracts from *Eugenia aurata* and *E. punicifolia* also inhibited inflammatory response by reducing neutrophil adhesion, degranulation, and NET release (55).

Classically PMA-mediated NOX-dependent ROS generation is mediated by the activation of protein kinase C (56, 57) while the NOX-independent pathway is mediated by calcium-activated small conductance potassium channel (SK3) and/or non-selective mitochondrial permeability transition pore (mPTP) in inducing mtROS production *via* intracellular Ca^2+^ flux (58, 59). Importantly, numerous reports have described the cross-talk of mtROS production and NOX activation representing a feed-forward vicious cycle of ROS production in oxidative stress (60). Douda et al. (2015) failed to observe cytoplasmic ROS generation *via* NOX in the presence of A23187 (58), while Vorobjeva et al. (2020), by using mitochondrial-targeted antioxidant and inhibitors of NOX, showed involvement of both NOX-derived ROS and mtROS in calcium ionophore-induced NETosis in human PMNs (59). We also observed a large amount of mtROS production by ionomycin during NOX-independent NETosis. We reported a significant reduction in the oxidative stress in GG-pretreated cells as demonstrated by its ability to suppress both ROS and mtROS. However, the anti-NETotic effect of GG was comparatively more pronounced in the case of ionophores than PMA. This indicates a putative role of GG as a mitochondrial-targeted antioxidant in preventing the mtROS–NOX feed-forward cycle and the ensuing oxidative insult and NETosis. Recently, Fortner et al. (2020) have also found that targeting mitochondrial oxidative stress with mitochondrial-targeted antioxidant MitoQ reduced neutrophil mtROS and NET formation in lupus-prone mice (61). Although our results point out mtROS as the prominent player, we cannot rule out the possibility of GG in reducing NETosis through other signaling pathways, particularly the NOX2–ROS axis, which requires further work to decode the detailed signaling pathways.

Instillation of Anu oil (from an Ayurvedic herb) exhibited reduced SARS-CoV-2 load and the expression of pro-inflammatory cytokine genes (Th1 and Th17) in the lungs (37). Recent reports have shown upregulation in IL-6 from human neutrophils using TLR8 ligand, resiquimod, and IFNα (62). NF-κB activation and the associated signaling seems to be the major pathways activated downstream of TRL7/8 activation leading to the release of pro-inflammatory cytokines. In one study using monocyte, Singh et al. (2015) had revealed enhanced ROS production and increased transcription, processing, and secretion of IL-1β upon activation of TLR-2 and -4 *via* cross-talk of p67phox-NOX2 with the IRAK–ERK pathway (63). The ROS-dependent pathway for the activation of inflammatory response *via* NOD-like receptor protein 3 (NLRP3) inflammasome has also been established (64, 65). Furthermore, multiple reports have suggested the involvement of NFκB in the regulation of the NLRP3 inflammasome (66). PAD enzymes are calcium-dependent enzymes, and PAD4 plays a prime role in ionomycin/A23187-dependent NETosis. Interestingly, PAD4 promotes NETs *via* regulating the assembly of NLRP3 inflammasome (67). Our results corroborate with this notion as A23187 showed significantly higher upregulation in IL-6 secretion as compared to PMA. Owing to the significant inhibition of ROS production, the anti-inflammatory effects of GG are apparent. The protective effect of GG on TRLM-primed inflammation showed a higher efficacy against calcium ionophore, further pointing out the critical role of NOX-independent pathways in GG-mediated regulation of different neutrophil functions. Although GG plays a role in the modulation of cytokine expression and its influence on NETs has been explored, the mechanism involved remains to be elucidated.

Besides NETosis to control the pathogen infection, these granulocytes have a plethora of other defense mechanisms. Being the most abundant leukocyte in blood, neutrophils are the prime phagocytes of the innate immune system (68). Modulation of innate immunity by medicinal plants or by their constituents has seen a renaissance in recent times. Our data did not reveal any major change in the phagocytosis by neutrophils when incubated with GG; however, other herbal plants from the same family reported an increase in the phagocytic activity of neutrophils, such as *Caesalpinia bonducella* and *Vigna mungo* (69, 70). Moreover, the intracellular bactericidal capacity of neutrophils was not affected by GG; a mild reduction probably suggests an inhibitory effect due to ROS/RNS production within the phagolysosomes during oxidative burst (71, 72).

In summary, here we provide the first direct evidence based on *in vivo* and *in vitro* data from hamster, mice, and human studies to show the potent protective efficacy of GG against COVID-19 pathologies and show that the mechanism of this protection could be mediated by strong anti-inflammatory response, inhibition of NETosis, and suppression of ROS generation.

## Methods

GG extract used in the study was prepared as per pharmacopeial standards and was provided by the National Medicinal Plant Board for the study.

### Animal ethics and biosafety statement

Six- to nine-week-old golden Syrian hamsters were procured from CDRI and quarantined for 1 week at a small animal facility (SAF), THST, before starting the experiment. A prophylactic GG group started to receive twice daily oral doses of GG 130 mg/kg (0.5% CMC preparation) 5 days prior to challenge and continued until end point. The remdesivir control group received 15 mpk sc injections of remdesivir 1 day before and 1 day after the challenge (23, 37). On the day of the challenge, the animals were shifted to ABSL3. Intranasal infection of live SARS-CoV-2 (SARS-Related Coronavirus 2, Isolate USA-WA1/2020) 10^5^ PFU/100 μl or with DMEM mock control was established with the help of a catheter under mild anesthesia by using a ketamine (150 mg/kg) and xylazine (10 mg/kg) intraperitoneal injection inside ABSL3 facility. All the experimental protocols involving the handling of virus culture and animal infection were approved by RCGM, institutional biosafety committee, and IAEC (IAEC/THSTI/105) animal ethics committee.

### Preparation of GG extract

GG extract was prepared by dissolving 10 mg of dry root powder of GG in 1 ml of water overnight in a shaker incubator at 37°C. The next day, the suspension was centrifuged at 10,000 × *g* for 30 min followed by filtration of the supernatant using a 0.45-μm filter. The filtrate was considered as 100% aqueous extract and diluted in water according to the experimental requirements.

### Virus culture and titration

SARS-Related Coronavirus 2, Isolate USA-WA1/2020 virus was grown and titrated in Vero E6 cell line cultured in Dulbecco’s Modified Eagle Medium (DMEM) complete media containing 4.5 g/L D-glucose, 100,000 U/L penicillin–streptomycin, 100 mg/L sodium pyruvate, 25 mM HEPES, and 2% FBS. The stocks of virus were plaque purified at the THSTI IDRF facility inside ABSL3 following institutional biosafety guidelines.

### Gross clinical parameters of SARS-CoV-2 infection

Post challenge, the body weight of the animals was recorded daily till 4 dpi. On 4 dpi, all animals were euthanized at ABSL3 and necropsy was performed to collect organs. Lungs and spleen of the animals were excised and imaged for gross morphological changes (23). The left lower lobe of the lung was fixed in 10% formalin and used for histological analysis (73). The remaining part of the lung left lobe was homogenized in 2 ml of Trizol solution for viral load estimation. Spleen was homogenized in 2 ml of Trizol solution. The Trizol samples were stored immediately at −80°C till further use. Serum samples isolated from blood were immediately stored at −80°C till further use.

### Viral load

Isolated lung was homogenized in 2 ml of Trizol reagent (Invitrogen) and RNA was isolated by the Trizol-Chloroform method. RNA yield was quantitated by nano-drop and 1 µg of RNA was used to reverse-transcribe cDNA using the iScript cDNA synthesis kit (BIORAD; #1708891) (Roche). cDNAs (1:5 dilution) were used for qPCR by using the KAPA SYBR^®^ FAST qPCR Master Mix (5X) Universal Kit (KK4600) on the Fast 7500 Dx real-time PCR system (Applied Biosystems), and the results were analyzed with SDS2.1 software (23, 37). Briefly, 200 ng of RNA was used as a template for reverse transcription-polymerase chain reaction (RT-PCR). The CDC-approved commercial kit was used for of the SARS-CoV-2 N gene: 5′-GACCCCAAAATCAGCGAAAT-3′ (Forward), 5′-TCTGGTTACTGCCAGTTGAATCTG-3′ (Reverse). The hypoxanthine-guanine phosphoribosyltransferase (HGPRT) gene was used as an endogenous control for normalization through quantitative RT-PCR. The relative expression of each gene was expressed as fold change and was calculated by subtracting the cycling threshold (Ct) value of the HGPRT-endogenous control gene from the Ct value of the target gene (ΔCT). Fold change was then calculated according to the formula POWER(2,-ΔCT)*10,000 (74, 75).

### qPCR from splenocytes

RNA isolated from spleen samples were converted into cDNA as described above. Thereafter, the relative expression of each gene was expressed as fold change and was calculated by subtracting the cycling threshold (Ct) value of the HGPRT-endogenous control gene from the Ct value of the target gene (ΔCT). Fold change was then calculated according to the formula POWER(2,-ΔCT)*10,000. The list of the primers is provided as follows.

**Table:** 

**Gene**	**Forward**	**Reverse**
HGPRT	GATAGATCCACTCCCATAACTG	TACCTTCAACAATCAAGACATTC
tryptase β2	TCGCCACTGTATCCCCTGAA	CTAGGCACCCTTGACTTTGC
chymase	ATGAACCACCCTCGGACACT	AGAAGGGGGCTTTGCATTCC
muc1	CGGAAGAACTATGGGCAGCT	GCCACTACTGGGTTGGTGTAAG
Sftpd	TGAGCATGACAGACGTGGAC	GGCTTAGAACTCGCAGACGA
Eotaxin	ATGTGCTCTCAGGTCATCGC	TCCTCAGTTGTCCCCATCCT
PAI-1	CCGTGGAACCAGAACGAGAT	ACCAGAATGAGGCGTGTCAG
IFNγ	TGTTGCTCTGCCTCACTCAGG	AAGACGAGGTCCCCTCCATTC
TNFα	AGAATCCGGGCAGGTCTACT	TATCCCGGCAGCTTGTGTTT
IL-13	AAATGGCGGGTTCTGTGC	AATATCCTCTGGGTCTTGTAGATGG
IL-17A	ATGTCCAAACACTGAGGCCAA	GCGAAGTGGATCTGTTGAGGT
IL-10	GGTTGCCAAACCTTATCAGAA ATG	TTCACCTGTTCCACAGCCTTG
IL-6	GGACAATGACTATGTGTTGTTAGAA	AGGCAAATTTCCCAATTGTATCCAG

### Histology

Fixed lung samples were embedded in paraffin blocks and sectioned into 3-µm sections mounted on silane-coated glass slides. The sectioned slides were then used for staining with hematoxylin and eosin dye as previously described (73). Each stained sample was then analyzed and captured at 10× magnification. Assessment for the histological score was carried out through blind scoring for each sample by a professional histologist on a scale of 0–5 (where 0 indicated the absence of histological feature while 5 indicated the highest score). Disease index score was calculated by the addition of all the individual histological scores.

#### *In vitro* differentiation of T cells

Single-cell suspension was prepared from spleen and lymph nodes of 6- to 8-week-old C57BL/6 mice. The cells were activated using soluble anti-CD3 (2 μg/ml) and differentiated into Th1 conditions by adding recombinant mouse IL-12 (15 ng/ml) cytokine, Th2 conditions by adding recombinant mouse IL-4 (15 ng/ml) cytokine, or Th17 conditions by adding TGF-beta (2 ng/ml) plus IL-6 cytokine (25 ng/ml) (75, 76). *Glycyrrhiza gabra* was added in concentrations ranging from 10 to 1,000 μg/ml at the start of culture. Cells were harvested after 72 h of culture. Intracellular cytokine staining was performed to check the expression of IFNγ, IL-4, and IL-17 cytokine for Th1, Th2, and Th17 cells, respectively.

#### Intracellular cytokine staining

Cells were stimulated for 4 h with PMA (phorbol 12-myristate 13-acetate; 50 ng/ml) and ionomycin (1.0 μg/ml) and a protein-transport inhibitor containing monensin before detection by staining with antibodies. Surface markers were stained for 15–20 min at room temperature in PBS with 1% FBS, then fixed in Cytofix and permeabilized with Perm/Wash Buffer using Fixation Permeabilization solution kit, and stained with anti-IL-17A, anti-IFNγ, and anti-IL-4 diluted in Perm/Wash buffer. All antibodies were used in 1:500 dilution. The cells were then taken for flow cytometry using BD FACSCantoII and data were analyzed with FlowJo software.

### Isolation of murine BMDNs and human peripheral neutrophils

Murine bone marrow-derived neutrophils were isolated from femur and tibia bones of C57BL/6 wild-type male mice (20–25 g, 12–16 weeks) using the method described previously (68). Long bones were flushed with HBSS + 0.1% BSA through a sterile tube followed by centrifugation at 400 × *g* for 10 min at 10°C. Pellets were resuspended in 45% Percoll and gently layered over Percoll density gradient (81%–62%–55%). After centrifugation at 1,700 × *g* for 30 min with an acceleration of 5 m/s^2^ and a deceleration of 4 m/s^2^, bands between 81% and 62% were harvested and assessed for their viability by Trypan blue and purity by anti-Ly6G and anti-CD11b antibodies.

Human neutrophils were isolated from the peripheral blood obtained from a healthy donor, by following the Percoll density gradient method as described earlier (77). Briefly, 2 ml of buffy coat was incubated with 6% dextran at 37°C for 30 min. The upper phase was collected and centrifuged at 1,800 × *g* for 10 min. Pellets were mixed in HBSS containing 0.1% BSA and layered on top of the Percoll gradient (81%–62%) before centrifuging at 1,800 × *g* for 30 min. The neutrophil-containing layer was collected, and the purity and viability of neutrophils were assessed by CD15 labeling and Trypan blue, respectively. All the studies on mice were approved by the institutional animal (THSTI/105) and human (THS1.8.1/100) ethical committees, DBT-THSTI, Faridabad.

### Cell viability assay

A total of 1.0 × 10^6^ per ml murine BMDNs and human PMNs were incubated with 100–1,000 µg/ml of GG for up to 240 min in flow tubes to assess the cytotoxicity of the herbal extracts as described earlier (78). For the cell death analysis, neutrophils were stained with cell-impermeant DNA intercalating propidium iodide (PI, 50 μg/ml) and were kept in the dark at RT for 15 min. DNA content was analyzed on a BD FACS Canto cell analyzer using BD FACS Diva software (BD Biosciences, USA).

### Intracellular ROS and mtROS analysis

Cytosolic and mitochondrial ROS was measured using the fluorescent probes DCFH-DA (10 μM) and MitoSOX (10 μM), respectively, in mouse BMDNs and human PMNs. A total of 1.0 × 10^6^ cells/ml were pre-incubated with GG before treatment with different interventions such as TRLM (10 μM), PMA (10–100 nM), A23187 (1–5 μM), ionomycin (1–4 μM), NAC (10 μM), and MitoTEMPO (10 μM). The control experiment contained the concentration of DMSO (0.1%) as in the extract-treated cells. A minimum of 10,000 events were acquired for each sample using BD FACS Canto II.

### NETosis assay

Neutrophils (5.0 × 10^5^ cells/ml) from mice or human were added to poly-L-lysine-coated wells and incubated with GG for 60 min at 37°C. SYTOX Green (100 nM) was added to each well, and cells were treated with TRLM (10 μM), PMA (10 μM), A23187 (1 μM), ionomycin (1 μM), VAS2870 (10 μM), Diltiazem (10 μM), or vehicle (DMSO 0.1%) and incubated in a CO_2_ incubator at 37°C. The fluorescence was measured at different time periods up to 240 min in a fluorescence plate reader at 37°C (Synergy 2; BioTek). The fluorescence of stimulated cells was expressed in arbitrary units and was determined by subtracting the baseline fluorescence of unstimulated cells from the activated cells. In a parallel experiment, immunofluorescence staining of BMDNs and PMNs was carried out using mouse anti-MPO and rabbit anti-H4Cit3 antibodies. After fixation and blocking, samples were incubated overnight with 1:100 dilution of primary antibodies and were visualized after incubation with the secondary antibodies (1:200, anti-mice AF488 and anti-rabbit AF594) using the confocal microscope (Olympus FV3000) at 100× resolution. DNA was stained with DAPI (0.5 μg/ml).

### Pro-inflammatory cytokine assay

The levels of IL-6 and TNFα in the supernatant/cell-free samples after the centrifugation were measured by using the monoclonal antibody-based mouse interleukin ELISA kits. Briefly, 100 μl of media from cells (1.0 × 10^6^ cells/ml) treated with TRLM and PMA/A23187, following pre-incubation with GG/vehicle, was used to measure the cytokines according to the manufacturer’s instruction.

### Phagocytosis and bactericidal assay

To assess the phagocytosis, PE-labeled latex beads were incubated with GG-pretreated human PMNs (1.0 × 10^4^) at a 1:50 (neutrophil:beads) ratio and were analyzed using FACS (72) after quenching the extracellular fluorescent signal of adherent beads with Trypan blue (0.4%). In a separate experiment, GG pre-exposed human neutrophils were treated with kanamycin-resistant *E. coli* for 30 min at 37°C. After lysis of the cells using sterile water, the diluted lysate was spread on LB agar plates with the antibiotic and kept at 37°C overnight. The bacterial colonies were counted and the bactericidal activity is expressed as a percentage of CFU in the presence and absence of neutrophils.

### Statistical analysis

All the experiments have been carried out independently in triplicate. Results are being expressed as mean ± SEM. Multiple group comparisons have been performed using one-way ANOVA followed by the Bonferroni test using GraphPad Prism 8. The differences have been considered as statistically significant when the *p*-value was < 0.05.

## Data availability statement

The datasets presented in this study can be found in online repositories. The names of the repository/repositories and accession number(s) can be found in the article/**Supplementary Material**.

## Ethics statement

The animal study was reviewed and approved by Institutional animal ethics committee (IAEC), THSTI.

## Author contributions

Conceived, designed, and supervised the study: MD and AA. Performed the experiments: ZR, PB, SS, and UM. ABSL3 experiment: ZR and SS. FACS: PB and UM. qPCR: ZR and PB. Viral load: ZR. Histology: ZR. ELISA: PB. Fluorescence microscopy: PB. Analyzed the data: ZR and PB. Contributed reagents/materials/analysis tools: MD and AA. Wrote the manuscript: ZR, PB, AA, and MD. All authors contributed to the article and approved the submitted version.

## Acknowledgments

MD and AA received financial support for this study from Ayush-DBT (BT/PR40378/TRM/120/486/2020 and NMPB/IFD/GIA/NR/PL/2020-21/53). AA and MD received financial support from THSTI Core to establish the hamster model for SARS-CoV-2 infection. We greatly acknowledge the support and critical inputs of Dr. Pramod Kumar Garg in the manuscript. We thank IDRF (THSTI) for the support at ABSL3 facility, Prabhanjan and Jitender for providing technical support, and a small animal facility and Immunology Core for providing support in experimentation. Hamsters were procured from CDRI, Lucknow. We thank ILBS for support in histological analysis and assessment. The following reagent was deposited by the Centers for Disease Control and Prevention and obtained through BEI Resources, NIAID, NIH: SARS Related Coronavirus 2, Isolate USA-WA1/2020, NR-52281.

## Conflict of interest

The authors declare that the research was conducted in the absence of any commercial or financial relationships that could be construed as a potential conflict of interest.

## Publisher’s note

All claims expressed in this article are solely those of the authors and do not necessarily represent those of their affiliated organizations, or those of the publisher, the editors and the reviewers. Any product that may be evaluated in this article, or claim that may be made by its manufacturer, is not guaranteed or endorsed by the publisher.
