# Supplementary figures and images for: Prophylactic treatment of Glycyrrhiza glabra mitigates COVID-19 pathology through inhibition of pro-inflammatory cytokines in the hamster model and NETosis
Source: Front Immunol. 2022 Sep 27;13:945583. doi: 10.3389/fimmu.2022.945583 (PMC9550929; doi:10.3389/fimmu.2022.945583)

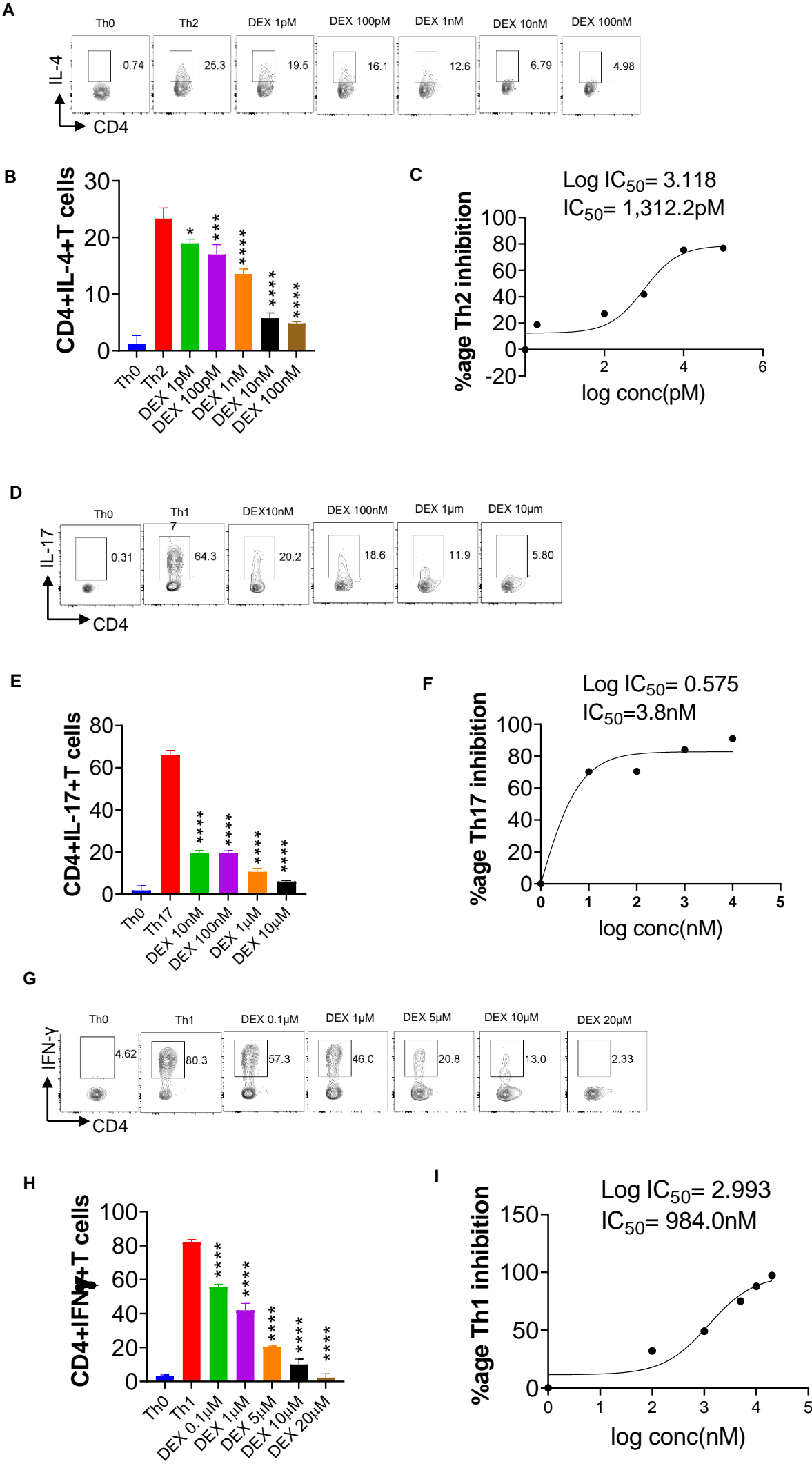

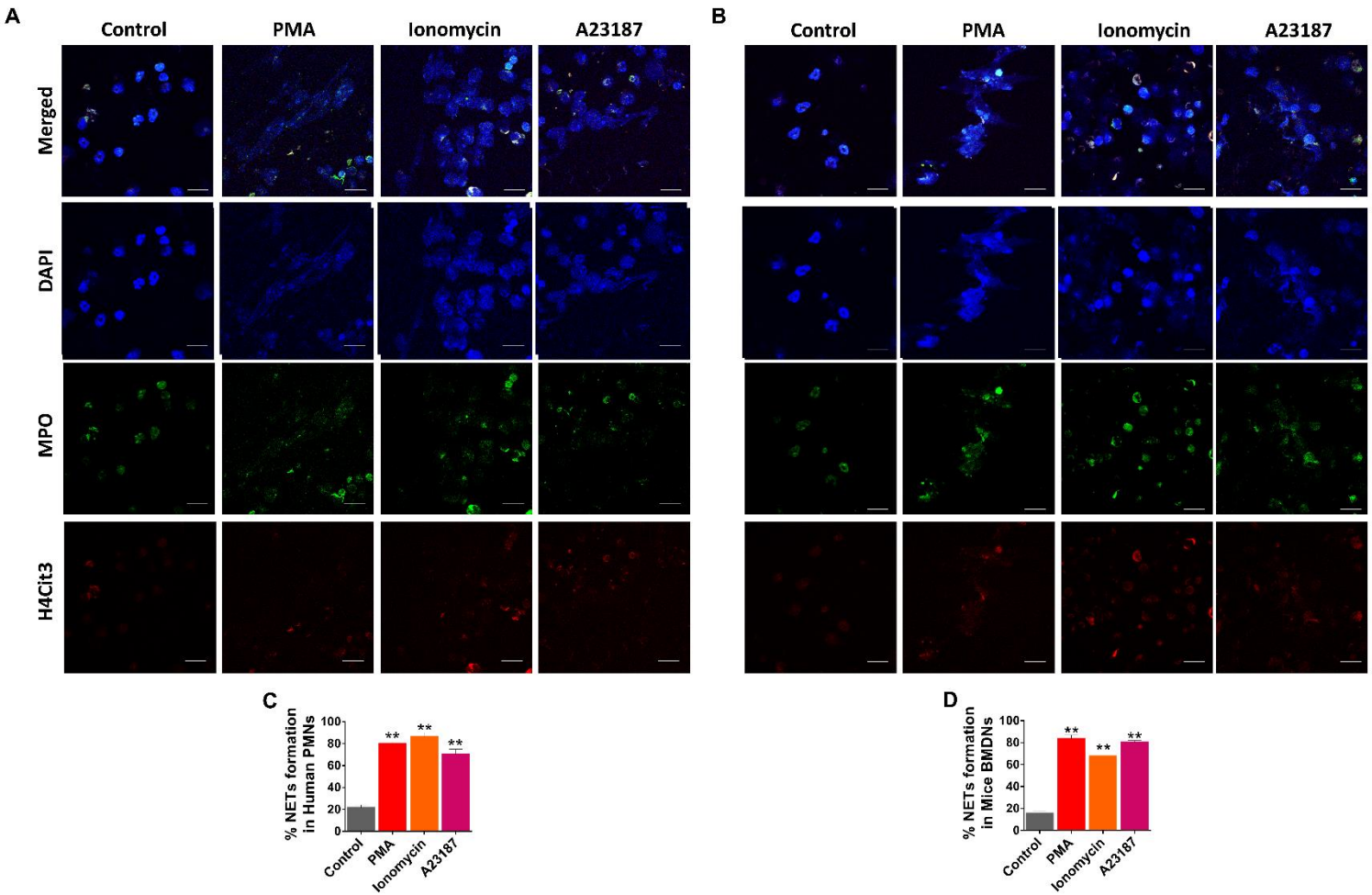

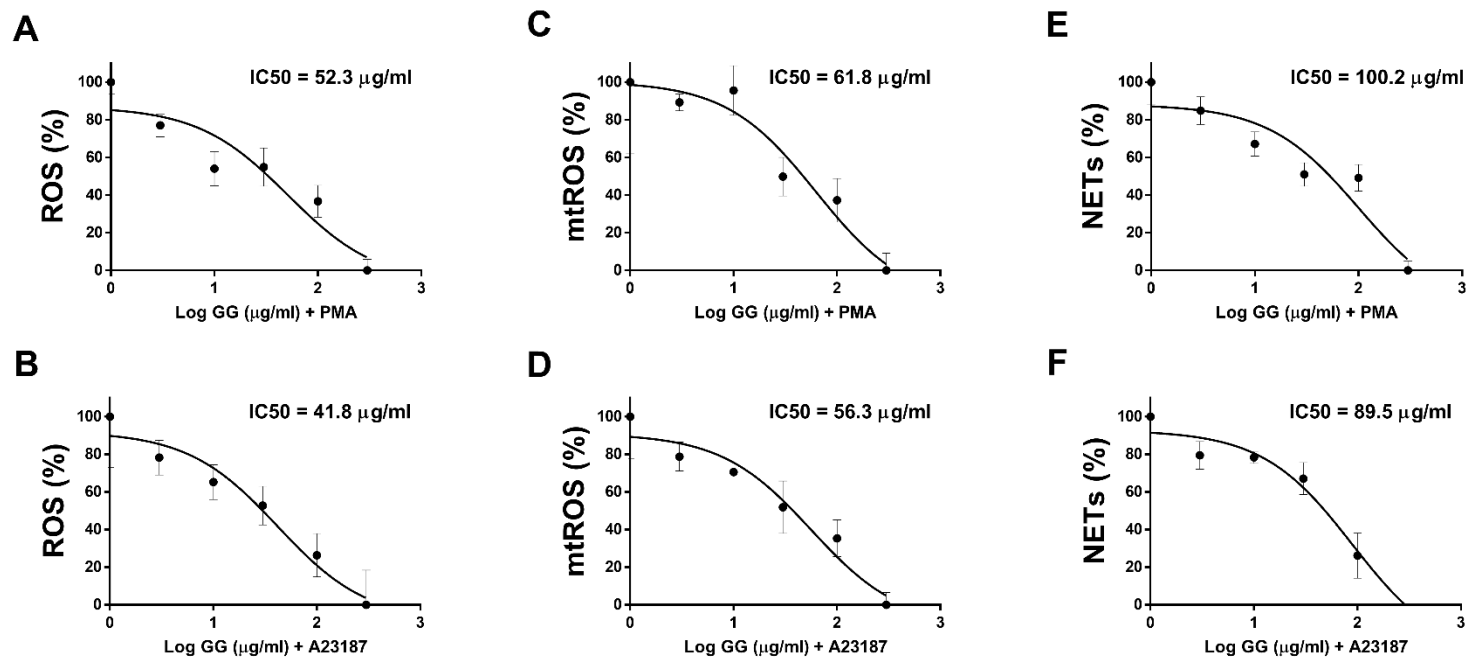

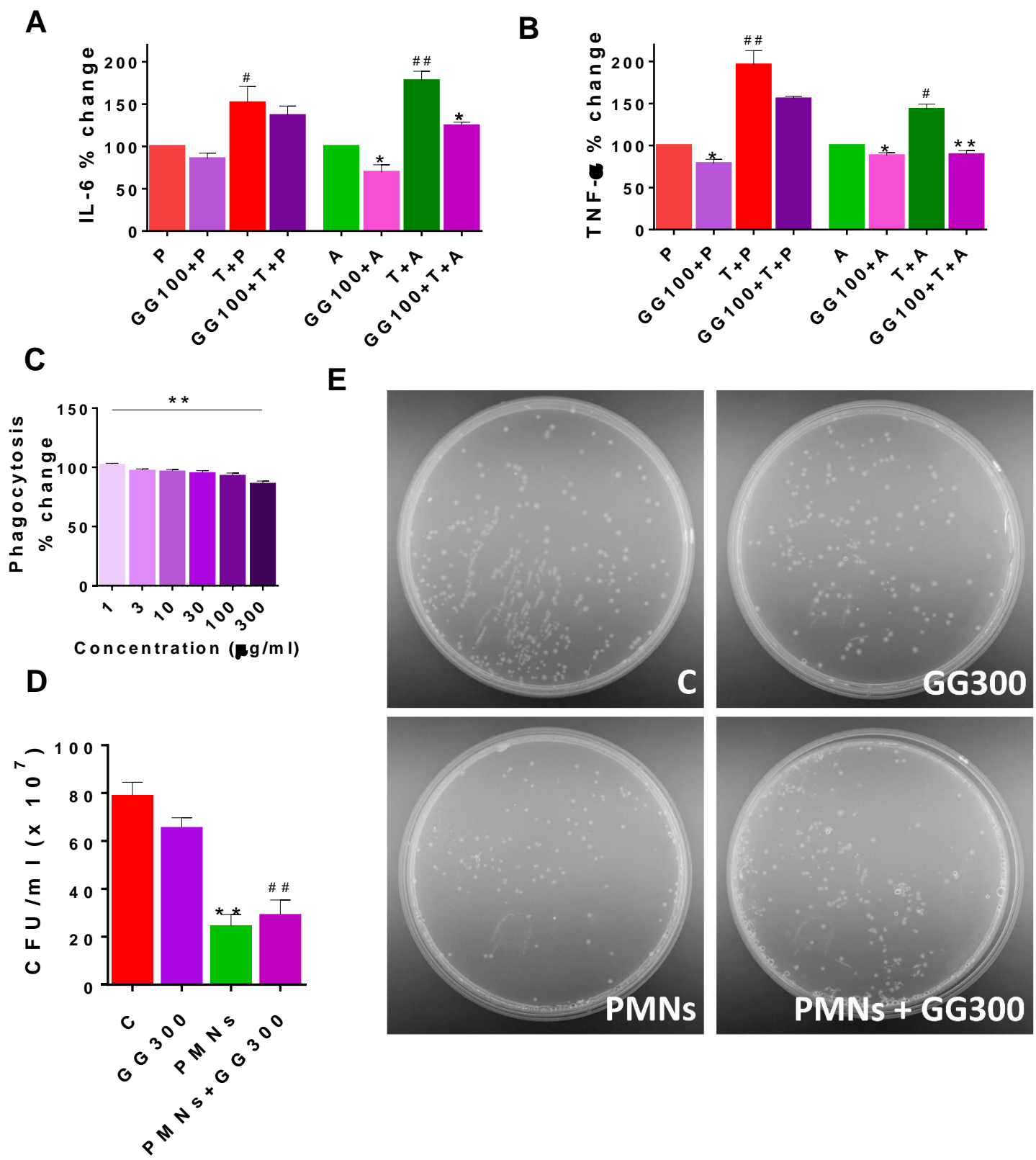

Supplement: Supplementary file 1 [file DataSheet_1.pdf]
